# Mercury Levels in Locally Manufactured Mexican Skin-Lightening Creams

**DOI:** 10.3390/ijerph8062516

**Published:** 2011-06-23

**Authors:** Claudia P. Peregrino, Myriam V. Moreno, Silvia V. Miranda, Alma D. Rubio, Luz O. Leal

**Affiliations:** Environmental Department, Advanced Material Research Center, Miguel de Cervantes 120, Chihuahua, Chih. 31109, México; E-Mails: claudia.peregrino@cimav.edu.mx (C.P.P.); myriam.moreno@cimav.edu.mx (M.V.M.); silvia.miranda@cimav.edu.mx (S.V.M.); alma.rubio@cimav.edu.mx (A.D.R.)

**Keywords:** mercury, whitening creams, atomic absorption, cold vapor

## Abstract

Mercury is considered one of the most toxic elements for plants and animals. Nevertheless, in the Middle East, Asia and Latin America, whitening creams containing mercury are being manufactured and purchased, despite their obvious health risks. Due to the mass distribution of these products, this can be considered a global public health issue. In Mexico, these products are widely available in pharmacies, beauty aid and health stores. They are used for their skin lightening effects. The aim of this work was to analyze the mercury content in some cosmetic whitening creams using the cold vapor technique coupled with atomic absorption spectrometry (CV-AAS). A total of 16 skin-lightening creams from the local market were investigated. No warning information was noted on the packaging. In 10 of the samples, no mercury was detected. The mercury content in six of the samples varied between 878 and 36,000 ppm, despite the fact that the U.S. Food and Drug Administration (FDA) has determined that the limit for mercury in creams should be less than 1 ppm. Skin creams containing mercury are still available and commonly used in Mexico and many developing countries, and their contents are poorly controlled.

## 1. Introduction

Mercury is well known for its toxicity. It is widely distributed in the environment by natural and anthropogenic sources. The major anthropogenic sources are mining, agriculture and industry [1]. There are however new, less explored routes of mercury exposure, such as its presence in cosmetics. Cosmetics are used on a daily basis with a good safety record, but make-up dyes containing highly toxic metals such as lead, mercury and cadmium oxides were in the cosmetic market until the early 20th century [2].

Currently, skin-spots represent an aesthetic concern in humans. This skin disorder is a consequence of an excess of melanin produced by hyperactivity of melanocytes, which are the cells responsible for skin pigmentation. This can be due to a variety of reasons, including overexposure to solar radiation, ageing, hormonal dysfunction during pregnancy or taking certain medicines [3]. This disorder can be reduced with cosmetic treatment through the use of so-called skin-whitening cosmetic products. These contain different chemicals, such as kojic dipalmitate (KDP), which produces a whitening effect on the skin, based on the inhibition of melanin biosynthesis via different mechanisms [4].

Despite the well-known hazards of mercury exposure, skin creams containing mercury are widely available in pharmacies and beauty aid stores in Mexico. The label on some of these products does not specify their ingredients, so the consumer does not have any choice for selecting suitable products. They are primarily used by women for their skin lightening effects [5–7]. A beauty cream named “Crema de belleza Manning”, produced and marketed in Mexico, had high mercury content. The analysis indicated that the skin cream contained 6 to 10 % mercury(I) chloride [8]. Its frequency of use had a seriously impact on the health of consumers, and its sale was banned permanently. Mercury salts inhibit the formation of melanin by competing with copper in the action of the enzyme tyrosinase [8]. While inadvertent oral ingestion is likely to be a more significant route of exposure, inorganic salts are easily absorbed through the skin and excreted through kidneys. This can result in skin changes such as facial burns and discoloration, among others [8,9].

A study of 119 Latino women from California, Arizona, New Mexico and Texas that were using bleaching creams to lighten skin tone, showed that 87% of them had elevated mercury levels in urine. Mercury concentrations in urine greater than 20 μg L^–1^ are associated with symptoms of mercury poisoning [8].

The Department of Health and Human Service (DHHS) and the International Agency for Research on Cancer (IARC) in the USA have not classified mercury as a carcinogen in humans, however, the EPA has determined that mercury chloride and methyl mercury are potentially carcinogenic in humans [10].

Al-Saleh *et al.* [11] analyzed several types of whitening creams from different countries, some of them containing high concentrations of mercury. In that study, the analyzed facial creams produced in Thailand, Lebanon and England contained the highest levels of mercury, ranging from 1,281 to 5,650 ppm. Uram *et al.* [12] have analyzed the mercury content of cosmetics made in Mexico. Some of them had the description of the ingredients on the label and others did not even have labels. It should be highlighted that the mercury concentration of “Blanca piel” cream (Ida Richtter, Mexico) was 1,325 ppm. This facial cream has been produced in Mexico, although it has been stated that it is a German formulation.

The US FDA lays the responsibility of checking the safety of their products and ingredients before introducing them to the market on the cosmetic firms. Most developing countries lack any safety regulations for cosmetics and other products that comply with the US FDA’s requirement such as labeling violations, the illegal use of color additives, and the presence of poisonous or deleterious substances such as pathogenic microorganisms [11]. The Mexican regulations established a list of prohibited and restricted substances in cosmetic formulations, which included mercury and its compounds as forbidden additives. Nevertheless, phenyl mercury and its salts as well as thimerosal, are allowed as cosmetic preservatives only in eye make-up to a maximum permissible mercury concentration of 0.007% (w/v) [13], whereas as color additives, mercury and its compounds are allowed up to 1 ppm [14].

Although the use of these products is harmful, their production and use continue, and it has become a global public health problem [9]. Due to this uncontrolled exposure, cosmetic products should be thoroughly evaluated for safety before marketing. Manufacturers and importers of cosmetics products should be required to generate a safety evaluation for each product including composition, specifications and final product evaluation [2,15,16].

There are several analytical techniques described in the literature for the determination of mercury in environmental and biological samples. However, the number of papers focusing on the sample pre-treatment and analysis of cosmetics is scarce [15].

The matrix of cosmetics is not simple; it usually contains many ingredients and often requires time-consuming and tedious sample treatments. Official analytical methods have been recommended in different legislations. Most of these methods use digestion or calcination as sample treatment and atomic spectrometry for determination. In the present study, we determined mercury content by cold vapor-atomic absorption spectrometry (CV-AAS) in different brands of facial cream samples. These samples were collected from various pharmacies and beauty aid stores in the Chihuahua market in order to check their safety and provide evidence of potential exposure to mercury poisoning. This could contribute to the reduction in the lack of regulatory inspection and to urge authorities to establish a control.

## 2. Materials and Methods

### 2.1. Reagents and Instrumentation

All solutions were prepared in Millipore-purified water (conductivity > 18.0 M·cm^–1^). All chemicals used were of analytical reagent grade and purchased from J.T. Baker. A 3 M·L^–1^ HCl (Instra-Analyzed, for trace metal analysis) was prepared daily from concentrated HCl acid and was used as carrier solution. The reducing solution was prepared by mixing 3 g of sodium tetrahydroborate and 3 g of sodium hydroxide in 500 mL Millipore water. Mercury standards were prepared by diluting a 1,000 mg·L^−1^ mercury(II) solution (National Institute of Standards and Technology, USA) in 2% HNO_3_. Glassware needed for mercury determination was soaked in 10% (v/v) HNO_3_ and rinsed with Millipore water. The reagents used for sample digestion were HNO_3_ (65% w/v, Suprapur) and hydrogen peroxide (30% w/v, analysis grade).

The acid digestion of samples was carried out in a microwave apparatus (CEM, model MARSx). The determination of mercury was carried out by two methods. One of the methods employed was the cold vapor generator (GBC, HG 3000) for analyzing low concentrations of mercury (CV-AAS). The samples with an elevated content of mercury were analyzed by Flame Atomic Absorption Spectrometry (FAAS), using a GBC avanta ∑ instrument.

### 2.2. Sample Preparation

A total of 16 skin-lightening creams were analyzed for determination of mercury. All available brands of whitening creams in pharmacies and beauty aid stores in the local market of Chihuahua were purchased (fourteen products). None of them indicated mercury content or had mercury indicated as an ingredient. The manufacturers were from several Mexican states (including Chihuahua) and one from Germany. Cream X and tonic X were brought by a beauty consultant, and both cosmetic products were unlabelled. Three replicates of each sample were prepared and analyzed. The creams tested are listed in Table 1.

Due to the unavailability of certified material for facial cream analysis, the accuracy of the method was determined by measuring two creams as reference materials, “Milagro whitening cream” (RM-1) and “Arambula bleaching cream” (RM-2), for which corresponding average mercury content were previously analyzed by other laboratories using AAS and ICP-OES (inductively coupled plasma-optical emission spectrometry). Fourteen samples of each reference material were analyzed. The average mercury concentration reported for RM-1 was 35,267 ± 787 ppm (n = 14), with a relative standard deviation (RSD) of 2.2%, whereas the average mercury content of RM-2 was 12,342 ± 435 ppm (n = 14) with a RSD of 3.5%. The sample pretreatment for reference materials as well as purchased samples was as follows: 0.25 g of sample was weighed and 8 mL of HNO_3_ and 2 mL of H_2_O_2_ were added. The mixture was subjected to a microwave digestion in two cycles: 35 min, 600 W, 200 °C and 10 min, 600 W, 100 °C. After cooling at room temperature, the sample was appropriately diluted with purified water. The absorbance of the aqueous matrix was measured in the AA apparatus.

## 3. Results and Discussion

### 3.1. Digestion Procedures

Preliminary studies were conducted in order to evaluate several digestion methods. The reference material RM-1 (“Milagro whitening cream”) was analyzed using four digestion procedures: HNO_3_ and a mixture of HNO_3_-H_2_O_2_ by conventional reflux digestion (CRD) and microwave-assisted digestion (MAD). The procedure for sample preparation has been described above. The recovery of mercury using HNO_3_ by CRD was 85%, whereas HNO_3_-H_2_O_2_ by CRD achieved 90%. This indicated considerable losses of mercury in the open digestion system. The MAD methods provided recoveries of 119% and 99% using HNO_3_ and HNO_3_-H_2_O_2_, respectively. The best results were obtained when the microwave-assisted digestion was based on sample dissolution with HNO_3_-H_2_O_2_ mixture. This method was selected for further experiments.

### 3.2. Analytical Parameters

The analytical curves were obtained with Hg(II) standards. The samples with an elevated content of mercury were analyzed by FAAS, obtaining linear calibration curves within the following ranges: 10–20, 20–50 and 80–150 ppm. Absorbance signals regarding the Hg solutions within each range were determined by carrying out cycles of three injections for each standard solution. The calibration graph within the range 80–150 ppm was evaluated for analytical purposes. Typical regression line between absorbance signal and mercury concentrations was described by the equation: Abs = 0.0265 + 0.002 *C*_Hg_ (ppm) with a correlation coefficient of 0.9998. The relative standard deviation (RSD), calculated from 10 successive measurements of 100 ppm Hg standard solution, was 0.94%. The CV-AAS was employed for analyzing low concentrations of mercury. The range of the linear calibration curve was 0.01–0.04 ppm. The equation was Abs = 0.0293 + 0.0041 *C*_Hg_ (ppm) with a correlation coefficient of 0.9984. The relative standard deviation (RSD) calculated from 10 successive measurements of 0.02 ppm Hg standard solution, was 2.8%. The detection limit achieved was 0.005 ppm. It has been calculated from three times the standard deviation of 8 blank signal measurements divided by the slope of the calibration curve (3*σ*_b_/*S*).

### 3.3. Validation of the Method

The accuracy of the technique was tested by the analysis of two whitening creams employed as reference materials and described in Section 2.2, *i.e.*, RM-1 and RM-2, which average mercury contents were 35,267 ± 787 and 12,342 ± 435 ppm, respectively. Three replicates of each sample were analyzed. The obtained values for Hg determination were 35,824 ± 1,639 for RM-1 and 12,035 ± 824 for RM-2, with Hg recoveries of 102% and 98%, respectively.

### 3.4. Mercury Content

The results of the mercury determination of the facial whitening creams is shown in Table 1. As can be seen, the mercury content of ten analyzed creams was less than the method’s detection limit using CV-AAS (0.005 ppm). However, the mercury content of the six other facial creams varied between 878 and 36,000 ppm. These values are extremely high and represent a serious health hazard. The mercury concentration was more than six times that found in other reports described in the literature, such as those reported by Al-Saleh *et al.* [11] and Uram *et al.* [12]. The production of the high mercury content creams was carried out in Mexico, except for the “Drula whitening cream”, which is made in Germany. The highest concentrations of mercury have been found in “Milagro” and “X” creams, both of unknown origin. On the label of “Milagro” it is stated that the product is made in Mexico, but the manufacturer and its address are not indicated.

Mexican regulations have been contravened, not only due to high mercury concentrations found in skin-lightening creams, but also by the labeling and marketing of these cosmetics products. Thus, the need of a regulatory inspection is mandatory. On the other hand, there is not an official report about mercury poisoning caused by the use of skin-lightening creams in Chihuahua, Mexico. Nevertheless, the symptoms of chronic mercury poisoning might be mistakenly misdiagnosed as another disease.

## 4. Conclusions

The overall results indicate that mercury content in whitening creams was extremely high in six analyzed samples and represents a serious health risk. According to the U S Food and Drugs Administration (FDA), cosmetic products should not contain mercury as an ingredient [17,18]. The mercury concentrations found in this study exceed any other report identified in our literature review. This might put consumers at the risk of mercury poisoning. Skin creams containing mercury are obviously still available and commonly used in the local marketplace and its contents are poorly controlled. To safeguard consumer health, our research calls for an immediate mandatory regular testing program to check mercury in whitening creams and other cosmetic products that are being marketed and consumed in Mexico.

Authors wish to thank Alejandro Benavides for technical assistance. Claudia P. Peregrino is grateful to the National Council of Science and Technology in Mexico (CONACYT) for the allowance of a scholarship. The financial support of CONACYT and The Government of Chihuahua State through project FOMIX CHIH-2008-C02-92172 is highly appreciated.

## Figures and Tables

**Table 1.** Mercury content in sixteen samples of Mexican whitening creams.

**Product name**	**Hg content (ppm ± SD)**	**Brand**
POND’S clarant B3 whitening cream	<0.005 ^c^	Unilever de Mexico
LUPITA cleansing cream	<0.005 ^c^	TELU
AVON whitening cream	<0.005 ^c^	Avon Cosmetics
CONCHA NACAR bleaching cream	<0.005 ^c^	Grisi Laboratories
WHITE SECRET whitening cream	<0.005 ^c^	Genomma Lab
BELLA AURORA whitening cream	<0.005 ^c^	Stillman Company Aurora
VITA NATURA whitening cream	<0.005 ^c^	Tunatural
BONAPIEL whitening cream	<0.005 ^c^	Forma Natura
FOREVER YOUNG bleaching	<0.005 ^c^	G+N Vida S.A.
TONICO X whitening lotion	<0.005 ^c^	Unknown
DRULA ^a,b^ whitening cream	878 ± 115	Drula Fabrik
SOMAR ROLF ^a^ whitening night cream	6,895 ± 1,305	Somar Laboratories
MYRYAM ^a^ whitening night cream	13,233 ± 279	Myryam Laboratories
ARAMBULA ^a^ bleaching cream	12,035 ± 824	Arambula Lab
CREMA X ^a^ whitening cream	19,882 ± 1,875	Unknown
MILAGRO ^a^ whitening cream	35,824 ± 1,639	Unknown

SD = Standard deviation;

a These creams were analyzed by Flame Atomic Absorption Spectrometry (FAAS), due to their high content of mercury (mg kg^−1^);

b This cream was produced in Germany;

c Less than the method’s detection limit (MDL).
